# ‘Get Healthy, Stay Healthy’: protocol for evaluation of a lifestyle intervention delivered by text-message following the Get Healthy Information and Coaching Service®

**DOI:** 10.1186/1471-2458-14-112

**Published:** 2014-02-04

**Authors:** Brianna Fjeldsoe, Philayrath Phongsavan, Adrian Bauman, Ana Goode, Genevieve Maher, Elizabeth Eakin

**Affiliations:** 1 School of Population Health, Cancer Prevention Research Centre, The University of Queensland, Brisbane, Australia; 2 Sydney School of Public Health, Prevention Research Collaboration, The University of Sydney, Sydney, Australia

**Keywords:** Extended contact, Maintenance, Sustained behaviour change, SMS, Telephone, Physical activity, Exercise, Diet, Weight loss, Intervention

## Abstract

**Background:**

Behavioural lifestyle interventions can be effective at promoting initial weight loss and supporting physical activity and dietary behaviour change, however maintaining improvements in these outcomes is often more difficult to achieve. Extending intervention contact to reinforce learnt behavioural skills has been shown to improve maintenance of behaviour change and weight loss. This trial aims to evaluate the feasibility, acceptability and efficacy of a text message-delivered extended contact intervention to enhance or maintain change in physical activity, dietary behaviour and weight loss among participants who have completed a six month Government-funded, population-based telephone coaching lifestyle program: the Get Healthy Information and Coaching Service (GHS).

**Methods/Design:**

GHS completers will be randomised to the 6-month extended contact intervention (Get Healthy, Stay Healthy, GHSH) or a no contact control group (standard practice following GHS completion). GHSH participants determine the timing and frequency of the text messages (3–13 per fortnight) and content is tailored to their behavioural and weight goals and support preferences. Two telephone tailoring calls are made (baseline, 12-weeks) to facilitate message tailoring. Primary outcomes, anthropometric (body weight and waist circumference via self-report) and behavioural (moderate-vigorous physical activity via self-report and accelerometer, fruit and vegetable intake via self-report), will be assessed at baseline (at GHS completion), 6-months (end of extended contact intervention) and 12-months (6-months post intervention contact). Secondary aims include evaluation of: the feasibility of program delivery; the acceptability for participants; theoretically-guided, potential mediators and moderators of behaviour change; dose-responsiveness; and, costs of program delivery.

**Discussion:**

Findings from this trial will inform the delivery of the GHS in relation to the maintenance of behaviour change and weight loss, and will contribute to the broader science of text message lifestyle interventions delivered in population health settings.

**Trial registration:**

ACTRN12613000949785

## Background

Overweight and obesity, along with insufficient physical activity and unhealthy dietary intake are associated with considerable burden of disease [1,2]. Over 60% of Australian adults are overweight or obese, 62% do not meet national physical activity guidelines and the vast majority do not meet dietary guidelines [2]. These population rates are similar to those in comparable developed countries [3-6]. While multi-sectoral approaches addressing policy, practice and social-environmental factors are needed to tackle obesity at a population level, there remains a need for effective broad-reach, individual-level interventions to support those adults who are currently overweight or obese (estimated nine million Australians) [7] to achieve and maintain moderate weight loss through health behaviour change.

Behavioural lifestyle interventions are effective at promoting initial weight loss [8,9]. However, maintaining improvements in these outcomes is often more difficult to achieve. Evidence indicates an average regain of 0.3 kg per month following the end-of-intervention [10,11], with the average participant regaining approximately 30% to 35% of their weight lost in the first year [12]. Within one [13] to five years [12,14] post intervention, 50% or more of participants are likely to have returned to their baseline weight. The challenge in maintaining weight loss post-intervention has been largely attributed to the failure in maintaining physical activity and dietary behaviour change [14,15].

Evidence from interventions designed to enhance the maintenance of physical activity, diet and weight loss suggests the importance of extended intervention contact after initial intervention [14,16,17]. Extended contact provides the opportunity to reinforce behavioural skills learnt during the initial intervention, support problem solving and provide continued accountability and motivation. A recent meta-analysis of randomised controlled trials (n = 11) of extended contact interventions for weight loss maintenance concluded that they are viable and efficacious [17]. Common features of successful extended contact interventions for weight loss maintenance include: contact from interventionists (rather than peers or non-interventionist contact); and, reinforcement of behavioural skills, particularly support for setting and meeting behavioural goals, problem-solving skills and training in relapse prevention [17]. Previous trials have primarily evaluated extended contact interventions delivered via face-to-face group or individual sessions [18-20], although it has also been shown that face-to-face session attendance decreases as treatment duration approaches one year and as individuals regain weight [14,21]. Some trials have found telephone-delivered extended contact interventions lead to better weight outcomes for participants compared to control groups [21-23]. There is mixed evidence supporting the efficacy of web-based extended contact interventions for weight loss and behaviour change [24], with poor results being attributed to the lack of active and ongoing engagement of participants with the website.

Mobile telephone text messaging may be particularly suited as a delivery modality for extended contact interventions. Text messages can: efficiently deliver tailored repeated contacts from interventionists; be actively “pushed” to participants to maintain contact over long periods of time; prompt behaviours and use of behavioural skills in real time; and, maintain two-way communication with an interventionist using minimal resources. Evidence is rapidly emerging supporting the efficacy of text message-delivered interventions to promote initial weight loss, physical activity and dietary behaviour change [25-30]. Recently, a small (n = 34), pilot trial reported continued weight loss from participants receiving a text message-delivered extended contact weight loss intervention [31]. This area of research holds great promise and requires ongoing investigation.

The Get Healthy Information and Coaching Service® (GHS) is a free, publicly available, telephone-delivered coaching program targeting healthy lifestyle improvements (moderate weight loss, physical activity and dietary behaviours) in adults [32]. The service was launched by the New South Wales Government in Australia in 2009 and since then three additional Australian states have taken it up. Evaluations of the GHS have shown weight loss and behavioural improvements at the end of the 6-month telephone coaching program [33] and evidence of maintenance 6-months after completion of the program for weight loss and some behavioural outcomes in a small sub-sample of participants [34].

This present study will test the feasibility and efficacy of a text message-delivered extended-contact intervention (Get Healthy, Stay Healthy; GHSH) in a randomised controlled trial, among GHS completers. As such, it will inform subsequent improvements to the GHS, in line with the New South Wales Ministry of Health’s commitment to evidence-based service delivery. Findings will also inform the broader field of interventions targeting maintenance of weight loss and multiple health behaviour change, particularly given the ‘real-world’ context of the evaluation and the potentially cost-effective means of intervention delivery. More specifically, in the trial we will assess the: 1) feasibility (intervention delivery and text message receipt tracking) and acceptability (participant satisfaction and engagement) of delivering the GHSH intervention; 2) efficacy of GHSH on changes in moderate-vigorous physical activity, fruit and vegetable consumption, body weight and waist circumference between baseline (at GHS completion) and 6-months (end of extended contact intervention) and 6-months and 12-months (end of maintenance phase); 3) mediators of change due to GHSH intervention (outcome expectancy, satisfaction with perceived outcomes, self-regulation, self-efficacy, social support and perceived environmental opportunity); 4) moderators of change due to GHSH intervention (demographics, health status, changes during initial GHS); 5) dose-responsiveness of GHSH intervention; and, 6) the costs to deliver the GHSH intervention.

## Methods

### Study design

This randomized controlled trial evaluates the GHSH extended contact intervention against a no-contact control group (standard practice following GHS completion). Participants are randomised following completion of the GHS. Data are collected at baseline (after GHS completion), 6-months (end of GHSH) and 12-months (6-months following GHSH completion). This trial commenced recruitment in August 2012 and is expected to be completed in June 2014. This study (main trial and the user testing pilot study) received ethical clearance from the Human Research Ethics Committee at The University of Sydney (Protocol No.: 03-2011/13523).

### Study context

The GHSH extended contact intervention was developed specifically to follow on from the GHS. The GHS is a publicly-available, lifestyle modification program involving ten telephone coaching calls (maximum 30 minute duration) over 6-months from a qualified health coach. The GHS is open to adults (age >18 years) who are at risk of chronic disease because they do not meet healthy eating [35] or physical activity guidelines [36] or they are overweight or obese. The majority of GHS clients are self-referred and contact the GHS via a free-call telephone number after having seen a GHS media advertisement. GHS clients can also be referred by health professionals and general practitioners, which triggers an outbound GHS call to invite them to participate. The GHS coaching calls aim to assist clients to develop skills in goal setting, maintaining motivation, overcoming barriers and making sustainable lifestyle changes [37]. GHS clients are able to tailor the focus of the coaching calls to be on one or all of the following areas: physical activity, healthy food choices and weight loss or maintenance. GHS clients have access to a website offering static educational content and paper-based behavioural and weight tracking tools.

### Participant recruitment

Participants for this study were recruited on a rolling basis from the pool of participants who completed the GHS between August 2012 and February 2013. Eligibility criteria for this study include: living in New South Wales, Australia; no intention of re-enrolling in GHS coaching; not involved in other GHS evaluation sub-studies; and ownership of a mobile telephone. During the recruitment timeframe, all GHS completers were invited to register their interest for the GHSH study by their GHS coach during the final coaching call. Verbal consent to contact was recorded and then GHS coaches emailed client contact details to the researchers. Interested participants were mailed a Participant Information Sheet and Consent Form and then contacted via telephone to establish their eligibility and willingness to consent to participate in the GHSH trial. Verbal consent to participation was audio recorded, and participants returned the signed Consent Form via reply paid post.

### Randomization

Once informed verbal consent was obtained, the participants underwent the GHSH baseline assessment and were randomized to one of the study groups. Participants were stratified based on their change in weight during the GHS (using the median GHS weight loss of 3 kg). Randomization was conducted using a randomization website (http://www.randomization.com), and allocation was conducted by a trained research assistant with no involvement in participant recruitment.

### Get Healthy, Stay Healthy intervention development and pilot testing

The GHSH intervention protocols were informed by a one-month user testing pilot study. Ten pilot participants were recruited during their final GHS coaching call and received an abbreviated user-testing version of the GHSH program (one telephone tailoring call, two weeks of tailored GHSH text messages). After the abbreviated program, participants completed a telephone interview to provide feedback on their experience. The majority of participants were female (8/10) and had lost between 2 to 15 kg body weight during the GHS. Overall, participants were surprised about how supported they felt during the two week text messaging period, with almost all expressing (unprompted) interest in continuing the program for 6-months if given the choice. On average participants opted to receive 10 text messages over two weeks (range 5–13 texts over two weeks). This selected frequency was above the expected rate, and during the follow-up interviews participants expressed that whilst they were happy with the self-selected frequency for the two week study, during a 6-month program they would select a lower frequency. Participants also stated that it was important that they could change the focus of their behavioural goals over time *“so it doesn’t get boring and repetitive”*. One participant did not like the tone of the language used in the text messages and found the phrasing *“patronising”*. The findings of this pilot study led to the following changes in the GHSH intervention protocol: participants can request changes to the text message frequency at any stage of the 6-month program; participants are prompted every 6-weeks to update their behavioural and weight goals; and, the language of the GHSH texts was revised to remove any overly directive terms.

### The Get Healthy, Stay Healthy (GHSH) intervention

**Figure 1:**
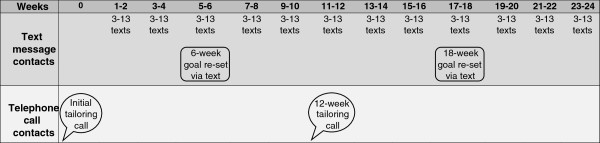
Overview of the Get Healthy, Stay Healthy intervention protocol.

The 6-month GHSH extended contact intervention is primarily delivered via text messages that are individually-tailored in terms of frequency, timing, content and wording. In order to tailor the text message content and to negotiate the participant’s text messaging preferences, participants receive two tailoring telephone calls; one call at the start of the intervention; and another call at mid-intervention (Figure 1). During the GHSH extended contact intervention participants choose whether they focus on a weight loss (no more than 2 kg per month) or weight maintenance goal and whether they focus on physical activity or diet or both behaviours (with targets consistent with national guidelines for physical activity and healthy eating [36,38]). This flexible, tailored approach to intervention targets is in line with the GHS coaching program.

#### Initial tailoring call

This 20–30 minute telephone call gathers information in order to tailor the text messages and is guided by a script. The call starts by reviewing what participants have achieved (in regards to their physical activity, diet and weight) during the GHS (this information is provided by the GHS coaching staff). Participants are asked to reflect on what they have achieved, what worked, and did not work to support them during their involvement in the GHS. They are guided to set two SMART (specific, measureable, achievable, realistic, time-based) goals for behaviour change (physical activity, diet or both) to achieve in the next 3-months. For each SMART goal, participants identify: a self-selected reward for reaching their goal; benefits they expect to experience if they reach their goals; preparatory behaviours to help reach their weekly goals; barriers and solutions to overcome them; and, a person who could support them to reach their goal and what specific action this person could take. Finally, participants select the number, timing and type of text messages they would prefer to receive for the first three months of the program. All of this information is recorded during the call and is used to tailor the content, timing and frequency of the GHSH text messages. In this study, the tailoring telephone calls are conducted by health coaches trained in behaviour change and motivational interviewing. However, ideally, when the GHSH extended contact intervention is taken up as part of the larger GHS; the GHS coach would conduct these two tailoring calls.

#### Get Healthy, Stay Healthy text messages

Participants select the number of text messages (within the range of three to 13 per fortnight), timing of texts (e.g. 6:00 am), and type of texts (e.g. optional texts about prompting preparatory behaviours). The texts reinforce behaviour change strategies discussed in the GHS (e.g., barrier identification, goal setting), as well as those shown in previous research to be important for maintaining behavioural changes: self-monitoring [10,39-41]; increased awareness of and satisfaction with positive outcomes of behavioural change [42-44]; environmental cues to behaviours [42,45,46]; and, self-regulation skills [47-49]. Wording of the text messages is tailored to individuals based on their: name, gender, long-term weight goals for next 3-months, short-term behavioural goals for next week, identified barriers and strategies to overcome them, preparatory behaviours to achieve their goals, perceived expectations of behavioural change and the name of a person who can provide them with social support to achieve their goals. All texts are signed off using the first name of the GHSH coach the participant spoke to during the initial tailoring call. GHSH text messages are limited to 160 characters. The content of the texts refers participants back to existing GHS infrastructure such as the paper-based weight tracker. The abbreviations used in the GHSH text messages are based on previous research with community samples on the comprehension of text message language [50].

There are four types of GHSH text messages that each target different behaviour change strategies and each have different minimum frequencies (Table 1). Firstly, all intervention participants receive a prompt to self-monitor their weight at least once per fortnight. Participants are not asked to report their weight via text message but simply prompted to weigh themself and record it in their GHS weight tracker. Second, participants receive a goal check text message at least once per fortnight that checks whether they met each of their behavioural goals in the past week. These goal check texts ask participants to reply ‘yes’ or ‘no’ to indicate their goal attainment. This reply option is voluntary and participants incur the costs of reply texts. Based on the participants’ reply they will receive a tailored goal check reply text message. These replies are often tailored to the participants’ anticipated outcomes of reaching their goals (see Table 1). The third type of text is a real-time behavioural prompt, which is optional and capped at a maximum of four per fortnight. These texts aim to remind participants of their SMART goals, preparatory behaviours and anticipated barriers and solutions to overcome them. The final type of GHSH text is sent once in Week 6 and Week 18 and it prompts participants to reflect on their weight and behavioural goals and re-set them appropriately. Participants are encouraged to let their GHSH coach know their new goals via reply text and these changes are reflected in subsequent text messages.

**Table 1:** Examples of the four types of Get Healthy, Stay Healthy text messages

**Text message type**	**Behaviour change strategies targeted**	**Example text messages**	**Minimum frequency**
**Self-monitoring of weight**	Self-regulation; Satisfaction with perceived outcomes	Take time today 2 weigh yourself Bob. It will help u c how ur tracking to lose another 2 kg & make u think about ur diet & exercise. Jenny	1 per fortnight
**Goal check of behavioural goals**	Self-regulation; Satisfaction with perceived outcomes	Hi Bob. Did u reach ur exercise goal 2 walk 30 mins x 4 times this week? Text me back yes or no so I know how ur going. Jenny	1 per fortnight for each behavioural goal
	Self-regulation; Outcome expectancy; Satisfaction with perceived outcomes; Self efficacy; Social Support	Congratulations on reaching ur weekly goal Bob. U need 2 reward ur efforts. Ur reward was 2 catch up with friends. Jenny	Only sent if participant responds to goal check
**Prompting behavioural cues**	Self-regulation; Perceived environmental opportunity; Self efficacy; Social Support	Bob u planned 2 walk today after work. Try putting ur exercise clothes on as soon as u get home & dont change until u have been 4 ur walk. Jenny	Optional (maximum 4 per fortnight)
		I know breakfast is hard 4 u 2 fit in. Set ur alarm 10 mins earlier or find a nutritious option 2 eat on the run. Bob this is important 4 ur health. Jenny	
**Goal re-set**	Self-regulation	Its important 2 re-set ur weight goal Bob. U currently want 2 lose another 2 kg. If u have a new goal 4 the next 6 weeks, reply & let me know. Jenny	Once in Week 6 and 18

Text messages are generated and sent by research staff, using a purpose-designed software package in which messages are able to be pre-programmed in advance and scheduled to be sent at specific times (e.g. Monday morning at 7 am). The software interfaces with a database of 115 pre-written text message frameworks, which each contain words and phrases that can be tailored based on the participant’s individual data. When developing these text message frameworks, each message was systematically mapped on to at least one of the targeted behaviour change strategies (as previously listed). Replies to the goal check texts are stored and the tailored response automatically triggered if the participants reply with the words ‘yes’ or ‘no’ (or accepted variations of these such as ‘nah’ etc.). If participants reply to a goal check with additional words (e.g. providing a justification as to why they did not meet their weekly goal) or a word not identified as a variant of ‘yes’ or ‘no’, then the program sends an email to research staff for them to manually decide which tailored goal check reply (met goal or did not meet goal) to send to the participant. Unprompted reply text messages from participants do not receive a reply and participants are made aware of this. At any stage the participants can change their text message preferences, behavioural or weight goals or withdraw from the study by contacting research staff via text message or telephone call. Any changes made to goals or text preferences are entered into the software program as soon as possible and subsequent texts incorporate these changes.

#### 12-week tailoring call

At three months (12-weeks) participants receive a telephone call to facilitate tailoring of texts based on updated information for the final three months of GHSH. The call is designed to last approximately 20 minutes and is guided by a script. This call follows a similar framework to the initial tailoring call and collects the same list of information for each of the two SMART goals (e.g. expected outcomes, barriers etc.). The protocol for this call stipulates that it should be made between Weeks 12 and 14, and if contact is not made during this period, the existing tailoring information is carried over for the final 3-months of the text messages.

### Control group treatment

The control group receives no contact, except for the evaluations at baseline, 6- and 12-months. Following completion of each assessment, control group participants are posted brief written feedback on their results to reduce attrition.

### Data collection

Data are collected from participants at baseline, 6-months (end of extended contact intervention) and 12-months (follow-up after 6-months of no contact). To enable comparison to outcomes from the broader evaluation of the GHS, the anthropometric and behavioural measurement tools used in this study are the same as those used in the GHS evaluation. In addition to these GHS-comparable measures, we are also collecting more detailed data on moderate-vigorous physical activity (via accelerometer) and dietary behaviours (via the Fat and Fibre Behaviour Questionnaire). Objective assessment of anthropometric outcomes (i.e. body weight, waist circumference) was not possible due to the cost of collection from study participants who could reside anywhere within the state of New South Wales.

At each assessment participants complete a computer-assisted telephone interview (CATI) conducted by a trained research assistant, fill out a paper-based questionnaire and wear an objective activity monitor. Participants in this study had previously completed pre- and post-GHS evaluations therefore, demographic data and data on change in primary outcomes during the initial GHS were not re-assessed in this protocol. The assessment tools employed are summarised in Table 2.

**Table 2:** Summary of outcomes and measures used in the Get Healthy, Stay Healthy trial

**Primary outcomes**	**GHS-comparable tools**	**Additional tools**
Moderate-vigorous physical activity (MVPA)	● 3-item physical activity assessment tool [51] capturing walking, moderate and vigorous activity (via CATI)	● Accelerometer (Actigraph GT1M, 10s epoch) capturing duration and frequency of MVPA
Dietary behaviours	● Two stand-alone items [56] on servings of fruit and vegetables per day (via CATI)	● Fat & Fibre Behaviour Questionnaire [58] via CATI (Total Index (1 to 5))
Body weight and waist circumference	● Self-reported (via CATI)	
**Secondary outcomes**	**GHSH-specific tools**	
Feasibility indicators	● Number and type of text messages sent	
	● Number and duration of tailoring interviews completed	
	● Number of prompted and unprompted text message replies from participants	
Acceptability indicators	● Recall of number of text messages received	
	● Treatment of text messages on receipt	
	● Categorical satisfaction ratings	
	● Qualitative feedback on intervention	
Mediators	● Outcome expectancy: MVPA [60]; diet [64]	
	● Satisfaction with perceived outcomes: MVPA and diet [59]	
	● Self-regulation: MVPA and diet [65]	
	● Self-efficacy: MVPA [66]; diet [67]	
	● Social support: MVPA and diet [68]	
	● Perceived environmental opportunity: MVPA [69]; diet [72,73]	
Moderators	● Demographics (e.g. age, education)	
	● Health status (e.g. chronic diseases, need for medical clearance before commencing GHS)	
	● Behavioural and anthropometric changes during initial GHS	

### Primary outcomes

#### Moderate-vigorous physical activity

At each assessment, participants complete a self-reported physical activity measure [51] during the CATI. This is a validated, 3-item assessment tool (3Q-PA) which asks participants to report the number of weekly sessions spent: walking for ≥30 minutes; doing moderate-intensity physical activity for ≥30 minutes; and, doing vigorous-intensity physical activity for ≥20 minutes. These sessions were summed to indicate the total number of health-enhancing physical activity sessions per week.

In addition to the self-reported measure, participants are posted a dual-axis accelerometer (Actigraph model GT1M; Actigraph, LLC, Fort Walton Beach, Florida), initialised to collect data in 10-second epochs, and a wear-time log. The wear log includes monitor fitting instructions and asks about any monitor removals, sleep time and whether the monitor was worn or removed during sleep. Before posting, research staff telephone the participant to negotiate a start date and reiterate the instructions from the wear-time log: to fit the accelerometer by elasticised band firmly around the waist at the right mid-axillary line; to wear the monitors continuously during all waking hours and to remove for any water-based activities that may damage the monitor. On the (expected) second day of wear, a follow-up telephone call is made by research staff to prompt participants to wear the monitor if they have not already done so and to return their accelerometer return when finished in a reply-paid envelope.

Accelerometer data are downloaded in Actilife (v 6.6.2) as both 10-second and 60-second epoch files and will be processed in SAS version ≥9.3 by a variety of methods. The main measures will be derived from the 60-second data using Freedson cutpoints (i.e., 1952 counts per minute [cpm] for moderate and 5724 cpm for vigorous) [52] as these are the most common of the validated approaches. Secondary approaches will include: a very low and very high cutpoint for moderate-vigorous activity [53,54] and Crouter’s two-step regression to ensure conclusions are robust to choice of cutpoint and use of a cutpoint-based versus variability-based approach. Analyses will be limited to days with ≥10 hours of wear and no excessive counts ≥20, 000 cpm (valid days). Bouts of ≥60 minutes of 0 cpm (allowing for < 3 minutes of counts 1–49 cpm) will be excluded as non-wear time [55]; self-reported sleep time will also be excluded for those wearing the monitor to bed. Outcomes used from the accelerometer data will include: average minutes per valid day of moderate-vigorous activity; and, average number of bouts per valid day of moderate-vigorous activity accumulated in ten minutes bouts (allowing for two one-minute epochs below this threshold).

#### Dietary behaviours

Participants are also asked the questions currently asked in the GHS evaluation via CATI. These questions ask participants to report their number of servings of fruit per day and vegetables per day [56]; and to report their average consumption of sweetened drinks per day (cups/day); and, takeaway meals per week (meals/week) [57].

Participants also complete the Fat and Fibre Behaviour Questionnaire (FFBQ) [58] during the CATI. This questionnaire assesses eating habits over the previous month. Nine items, scored from 1 (‘6 or more days per week’) to 5 (‘Never’), relate to consumption of particular high-fat or high-fibre foods. The remaining items, scored from 1 (‘Never’) to 5 (‘Always’), ask about behaviours related to cooking, eating or choice of foods. The three indices from the FFBQ (Total Index (20-items), Fat Index (13-items) and Fibre Index (7-items)) were responsive to change in our previous trials [59]. The indices have good two-week test-retest reliability (ICC = 0.87- 0.89), Pearson’s correlations of 0.50-0.56 with fat and fibre intake assessed by Food Frequency Questionnaire and similar or higher responsiveness compared to the Food Frequency Questionnaire [58]. The primary index of interest for this study will be the Total FFBQ Index (range 1–5).

#### Anthropometric outcomes

Body weight and waist circumference are self-reported by participants at each assessment point during the CATIs. Participants are asked to report their body weight in kilograms, measured whilst wearing light clothes and no shoes. They were encouraged to weigh themselves at the time of the interview if scales were present; otherwise they were asked to report their most recent weighing. Participants also report their waist circumference in centimetres during the CATI. Participants are posted a measuring tape and instruction sheet (with images) at baseline. The CATI interviewer instructs participants to take this measurement during the call, guiding them to measure from the top of their hip bone and to keep the tape straight. A validation sub-study of GHS participants (n = 38) revealed that self-reported weight was 1.6 kg (95% CI: 0.8 to 2.4 kg) lower than objectively measured weight [33]. There was 87% agreement between self-reported and objectively measured waist circumference classifications [33]. Body Mass Index (BMI) will be calculated based on participant’s self-reported height at baseline and their self-reported weight at each assessment point.

### Secondary outcomes

#### Feasibility outcomes

Tracking data from our purpose-designed text message software will provide data on intervention delivery (i.e., number and type of text messages sent) and intervention engagement from participants (i.e. prompted text message replies from participants, unprompted text messages from participants, proportion of goal checks responded to and achievement of weekly behavioural goals). The number and duration of tailoring interviews attempted and completed is also tracked.

#### Acceptability outcomes

Data collected via a self-completed paper questionnaire at six months will assess participants’ recall of the number of texts received in the past week, their treatment of text messages after receipt (read and stored/read and deleted/deleted without reading) and categorical ratings of satisfaction (five categories ‘not at all satisfied’ to ‘extremely satisfied’) with GHSH and ratings of usefulness (five categories ‘not at all useful’ to ‘extremely useful’) for GHSH overall and specifically for support for achieving their behavioural and weight loss goals. In addition, participants are asked to write a one sentence, qualitative description of the GHSH intervention and are invited to complete a telephone interview (approximately 10 minutes) involving open-ended questions regarding intervention usage, satisfaction and potential program improvements.

#### Proposed mediators

The proposed mediators relating to the skills targeted in the GHSH intervention are measured at each assessment via paper-based questionnaire.

*Outcome expectancy for physical activity:* is assessed using participants ratings on a five-point Likert scale (1 = ‘strongly disagree’; 5 = ‘strongly agree’) of the likelihood of seven positive outcomes of regular physical activity (i.e., more energy; improved mental wellbeing; lower stress levels; increased confidence; feeling good immediately after exercise) and four negative outcomes (i.e., possible injury; sore muscles or joints; feeling tired; having less time to do other things). As suggested by Rodgers and Brawley [60], the specific physical activity outcomes were determined from previous cross-sectional evidence on the common positive outcomes [61] and negative outcomes [62,63] of regular physical activity.

*Outcome expectancy for diet:* items were adapted from Zunft and colleagues [64] who reported on the main perceived benefits of healthy eating among European adults. The negative outcome expectancies for diet were based on previous qualitative studies on perceived barriers and outcomes of weight loss [62,63]. Participants rate on a five-point Likert scale (1 = ‘strongly disagree’; 5 = ‘strongly agree’) the likelihood of five positive outcomes of healthy eating (i.e., weight loss; better control over weight; prevention or control over diseases; more energy; improved physical health) and four negative outcomes (i.e., feeling hungry; costing more; missing out on my favourite foods; not feeling comfortable in social situations).

*Satisfaction with outcomes for physical activity and diet:* based on the methods of Courneya and colleagues [59], this study uses one question for each outcome expectancy item to explicitly measure satisfaction of experiencing each physical activity outcome (e.g., I am satisfied that my current level of exercise improves my mental wellbeing) and dietary outcome (e.g., I am satisfied that my current diet has helped me to lose weight). The response scale consists of a five-point Likert scale (1 = ‘strongly disagree’; 5 = ‘strongly agree’).

*Self-regulation for physical activity and diet:* are measured using 20 modified items from Petosa’s [65] original 43-item questionnaire. This original questionnaire assesses the use of self-regulation strategies to support physical activity adoption and maintenance [65] and has been used successfully to detect change in physical activity self-regulation (overall α = 0.88; subscales α = 0.82 – 0.96). Self-regulation for diet is measured using a scale of 19 items that were also adapted from Petosa [65] covering self-monitoring, goal setting, reinforcement, time management, and relapse prevention. Each item asks how often the strategies were used in the last month on a five-point Likert response scale (1 = ‘never’; 5 ‘very often’).

*Self-efficacy for physical activity:* is measured using a 5-item scale with good internal consistency (α = 0.82) [14] and two-week test-retest reliability (r = 0.90) [66]. Seven items asks about confidence to engaging in regular physical activity under different scenarios (from 1 = ‘not at all confident’ to 5 = ‘extremely confident’).

*Self-efficacy for diet:* is measured across seven items with an adapted version of a diet barrier self-efficacy scale [67] that asks about confidence to eat a healthy diet under different scenarios (from 1 = ‘not at all confident’ to 5 = ‘extremely confident’).

*Social support for physical activity and diet*: is measured using a shortened version of the Social Support for Diet and Exercise Scale [68]. The original measure has good test retest reliability (r = 0.55 to 0.86, p < 0.001) and internal consistency (α = 0.61 to 0.91) [68]. The modified version includes 10 items each for diet and physical activity regarding frequency of support received in the past month (1 = ‘never’ to 5 = ‘very often’) from friends, family, or household members.

*Perceived environmental opportunity for physical activity:* are measured using an 8-item version of the International Prevalence Study on Physical Activity – Environmental Module [69]. The questions were modified slightly to suit the Australian population (e.g., sidewalks are now referred to as footpaths). Participants respond to statements about: access to shops, public transport and recreational facilities; presence of footpaths; crime and traffic safety; social environment; and, aesthetics of their neighbourhood (defined as 10–15 minute walk from home) on a five-point scale (1 = ‘strongly disagree’; 5 = ‘strongly agree’). This scale has shown good test retest reliability in Swedish [70] and Japanese [71] samples.

*Perceived environmental opportunity for diet:* The 8-item measure used in this study was modelled on the existing measures of food choice and affordability [72] and perceived availability of healthy foods in neighbourhood items [73], with additional items that evaluated neighbourhood takeaway perceptions. Neighbourhood is defined as 10–15 minute walk from the participant’s home and the response scale consists of a five-point scale (1 = ‘strongly disagree’; 5 = ‘strongly agree’).

#### Moderators

Proposed moderators of intervention outcomes that will be considered are: demographic characteristics (i.e. age, gender, education status, relationship status, cultural background, employment status); health status (i.e. chronic disease diagnoses prior to GHS enrolment, need for medical clearance for GHS enrolment); pre-intervention weight loss and behaviour changes (i.e., during the initial GHS); and, self-selected dose of GHSH text messages (including total frequency and frequency by types of text messages).

#### Costs of intervention delivery

These costs are tracked systematically in terms of personnel time and direct delivery costs. Costs are tracked for all intervention-related tasks, including: sending text messages; conducting tailoring calls (call costs and personnel time for preparation, attempts and successful calls); entering data into software for tailoring text messages; and, manually triggering replies to behavioural goal checks that are not recognised by the software.

### Statistical analysis

#### Intervention effects

Data will be analysed using intention-to-treat principles (i.e., participants will be analysed according to their randomly assigned group, regardless of the amount of intervention received). Each primary outcome will be modelled using mixed linear models with random intercepts, the fixed effects of study group, time (6-months/12-months) and a group by time interaction and will adjust for baseline values and potential confounders. Depending on the distribution of the continuous, interval or categorical outcome an appropriate distribution (e.g. normal, log-normal, gamma, negative binomial, binomial) and link (e.g., identity, logit) will be used. These models will assess intervention effects at end-of-intervention (6-months), end-of-maintenance (12-months) as well as differences between end-of-intervention and end-of-maintenance. Potential confounders include baseline demographic, behavioural and health characteristics as well as pre-intervention weight loss and behaviour changes. Those variables associated with the outcome at p < 0.2 will be adjusted as potential confounders. Final models will include all these potential confounders or remove those that do not affect estimates to within ±20% if models show evidence of overfitting. Sensitivity of conclusions to assumptions regarding missing data will be evaluated by comparing results obtained using different techniques of handling missing data (completers analysis, covariate adjustment and multiple imputation).

#### Secondary analyses

Feasibility and acceptability outcomes will be reported descriptively. Mediation and moderation analyses will be exploratory. The extent to which theoretically-driven constructs and mechanisms for behaviour change mediate the intervention effects will be examined using a simple product-of-coefficient approach using Sobel tests [74]. Point estimates and bootstrap confidence intervals of path coefficients and the product of the mediated path coefficients will be used to determine the potency, certainty and direction of any mediation effect [74]. Moderator analysis will examine whether variation of intervention effects differ across demographic characteristics, health status and pre-intervention weight loss/behaviour change (i.e. during initial GHS). These analyses will test intervention effects (as above) but using interaction terms to allow the effects of group, time and group by time to also vary by each moderator. These models will assess moderation of end-of-intervention, end-of-maintenance as well as differences in moderation between these periods.

### Sample size

The estimated differences between groups in change in primary outcomes (and standard deviations) for this trial are based on the outcomes from the maintenance evaluation of the GHS from 6 to 12 months without ongoing contact (same as control group treatment in the current study) [34] and literature on change in anthropometric and behavioural outcomes during extended contact interventions [17,18,31]. This evidence shows that we can expect small, continued improvements in the GHSH group from baseline to 6 months and declines in the no contact control group. Based on this, the minimum differences of interest in changes between groups from baseline to 6 months for our primary outcomes are: 2 sessions/week of self-reported moderate-vigorous physical activity; 1 serve of fruit per day and 1 serve of vegetables per day; 2 kg body weight; and, 4 cm waist circumference. The sample size requirement was largely determined by self-reported physical activity (sessions/week) as this outcome required the largest number of participants. Allowing for an attrition rate of 20%, the targeted sample size of 106 participants per group (212 in total) is needed to provide a ≥90% power with 5% significance (two-tailed) to detect a between group difference in change of 2 sessions per week of moderate-vigorous physical activity (assuming a standard deviation (SD) of 5.7sessions/week [34] and pre-post correlation (r) of 0.70 [unpublished data]). It is estimated, based on previous evaluations of GHS [34, unpublished data], that this sample size also provides ≥90% power to detect minimum differences of interest in all other primary outcomes including fruit serves/day (assumed SD = 0.9, r = 0.40), vegetable serves/day (assumed SD = 1.5, r = 0.37), weight in kilograms (assumed SD = 16.5, r = 0.97) and waist circumference in centimetres (assumed SD = 13.5, r = 0.80); as well as our additional measures of physical activity (accelerometer-measured moderate-vigorous physical activity minutes/week (assumed difference = 60, SD = 147, r = 0.60) [75]); and dietary behaviours (FFBQ Total Index (assumed difference = 0.20, SD = 0.50, r = 0.70 [76]).

## Discussion

Extended contact following behavioural weight loss interventions is considered best practice for the maintenance of weight loss [17]. Text messaging may offer an ideal medium to deliver this extended contact. The Get Healthy, Stay Healthy (GHSH) trial will evaluate the feasibility, acceptability, efficacy, mediators and moderators of weight loss maintenance and behaviour change of a text message-delivered extended contact intervention following completion of the GHS - a population-based telephone-delivered healthy lifestyle program. A recent review of the evidence for extended contact interventions [17] recommended that future research identify dose-responsiveness of extended contact, exploit the strengths of mediated delivery modalities (e.g. real-time prompting by handheld devices), and examine the maintained effect of extended contact. The present study is responsive to this call and will also examine dose-responsiveness, maintenance and report on intervention delivery costs.

In the broader context of population-based weight loss and behaviour change programs (and in a context of scarce health care resources) funding agencies have practical questions that go beyond intervention effectiveness, including: How long do effects last and for how long it is necessary to deliver the intervention?; For whom does the intervention work?; and, Could it be delivered in a shorter and/or more cost-effective format? There is a paucity of research that speaks to these issues, all of which are important in informing uptake, implementation and sustainability of such programs delivered in real world, up-scaled population health contexts. Our research partners, the New South Wales Ministry of Health, recognise the critical importance of maintaining program outcomes to validate their ongoing expenditure on the GHS. The current study, with its rigorous methodology, has the capability not only of informing future service delivery of the GHS but also contributing to the broader evidence base on cost-effective means of promoting weight loss maintenance and multiple health behaviour change.

## Abbreviations

GHS: Get Healthy Information and Coaching Service; GHSH: Get Healthy, Stay Healthy; CATI: Computer-assisted telephone interview; FFBQ: Fat and Fibre Behaviour Questionnaire.

## Competing interests

The authors declare that they have no competing interests.

## Authors’ contributions

BF conceived of the study, contributed to the design of the study, drafted the manuscript and approved the final manuscript. PP contributed to the design of the study and approved the final manuscript. AB contributed to the design of the study and approved the final manuscript. AG contributed to the design of the study and approved the final manuscript. GM coordinated participant recruitment and data collection, contributed to the design of the study and approved the final manuscript. EE conceived of the study, contributed to the design of the study and approved the final manuscript.

## Pre-publication history

The pre-publication history for this paper can be accessed here:

http://www.biomedcentral.com/1471-2458/14/112/prepub
